# Bidirectional Decoupled Distillation for Heterogeneous Federated Learning

**DOI:** 10.3390/e26090762

**Published:** 2024-09-05

**Authors:** Wenshuai Song, Mengwei Yan, Xinze Li, Longfei Han

**Affiliations:** School of Computer and Artificial Intelligence, Beijing Technology and Business University, Beijing 100048, China; sws123258@gmail.com (W.S.); yandreamw@gmail.com (M.Y.); smilewhenever777@gmail.com (X.L.)

**Keywords:** information theory, personalized federated learning, knowledge distillation

## Abstract

Federated learning enables multiple devices to collaboratively train a high-performance model on the central server while keeping their data on the devices themselves. However, due to the significant variability in data distribution across devices, the aggregated global model’s optimization direction may differ from that of the local models, making the clients lose their personality. To address this challenge, we propose a Bidirectional Decoupled Distillation For Heterogeneous Federated Learning (BDD-HFL) approach, which incorporates an additional private model within each local client. This design enables mutual knowledge exchange between the private and local models in a bidirectional manner. Specifically, previous one-way federated distillation methods mainly focused on learning features from the target class, which limits their ability to distill features from non-target classes and hinders the convergence of local models. To solve this limitation, we decompose the network output into target and non-target class logits and distill them separately using a joint optimization of cross-entropy and decoupled relative-entropy loss. We evaluate the effectiveness of BDD-HFL through extensive experiments on three benchmarks under IID, Non-IID, and unbalanced data distribution scenarios. Our results show that BDD-HFL outperforms state-of-the-art federated distillation methods across five baselines, achieving at most 3% improvement in average classification accuracy on the CIFAR-10, CIFAR-100, and MNIST datasets. The experiments demonstrate the superiority and generalization capability of BDD-HFL in addressing personalization challenges in federated learning.

## 1. Introduction

In the age of big data, protecting data privacy is becoming increasingly important. Machine learning (ML) relies on high-quality datasets to create unbiased and generalizable models. However, implementing and maintaining such data is challenging due to technical and regulatory barriers [1]. Additionally, the inability to collect large amounts of data on a centralized server poses significant privacy challenges for deep learning.

To solve the data privacy problem, a decentralized privacy-preserving solution known as federated learning has emerged, which helps us mitigate the “data silo” dilemma by allowing client devices to complete each training procedure independently. Subsequently, the trained models are uploaded to the server, aggregated, and distributed globally [2,3]. Despite its advantages in data privacy, FL faces a significant challenge that impacts its performance: statistical diversity. Specifically, the Heterogeneous Federated Learning (HFL) models are trained on the device using locally available data. However, data across different devices often vary in distribution, encompassing different types and imbalanced quantities. This heterogeneity leads to models optimizing in different directions during training. As a result, when the global federated model is aggregated from these local clients, it often struggles to generalize effectively across individual devices, resulting in insufficient local personalization.

**Figure 1:**
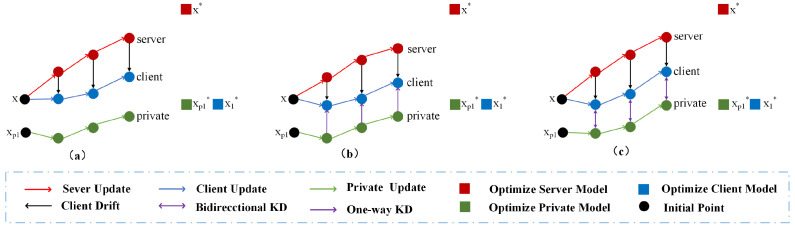
Changes in optimization direction of models (server, client, and private) for distillation methods in Heterogeneous Federated Learning and normal federated learning. The server is constantly updated toward optimal point $x^{*}$ (red square), the client toward optimal point $x_{1}^{*}$ (blue square), and the private model toward optimal point $x_{p1}^{*}$ (green square). The server and the client start from the same starting point *x* of the function f(x), and the private model starts from point $x_{p1}$. (**a**) In standard federated learning, cross-entropy optimization is applied without any knowledge exchange among clients. This approach results in a loss of personalization due to data heterogeneity across different clients. (**b**) The one-way distillation method, which utilizes relative-entropy loss, allows the local model to distill knowledge from the private model. However, this approach causes the private model to lose access to global information from the aggregated server model, impairing its generalization capability. (**c**) In contrast, bidirectional distillation with decoupled relative-entropy optimization enables mutual knowledge transfer between the private and local models. This approach allows both models to learn global information, thereby maintaining generalization while preserving local personalization.

To solve the problem of local personalization, many Personalized Heterogeneous Federated Learning (pHFL) methods have been proposed, which can be divided into three categories: (1) the global fine-tuning model, exemplified by FedRep [4] and Per-FedAvg [5], which uses the global model for fine-tuning to obtain the client models; (2) the local personalized aggregation model, typified by PartialFed [6], APPLE [7], FedALA [8], and FedAF [9], which leverages client relationships to improve personalized model performance; and (3) the personalized private model, including FedMD [10], FML [11], pFedMe [12], and Ditto [13], which increases the level of personalization by introducing additional models in clients. However, the global fine-tuning model solely relies on a single global model for fine-tuning client models as shown in Figure 1a. In practical applications, the model structure of each client differs, which restricts local personalization [14]. The local personalized aggregation model improves personalization capabilities efficiently. However, similarity metrics, such as clustering or unsupervised technologies [15] on clients are challenging, and the associated computational cost is high.

In contrast, the personalized private model achieves local personalization by introducing additional models, which leverages knowledge distillation to overcome the restriction imposed by heterogeneous clients. FedMD, FML, or pFedMe distills knowledge from the private model to the local model using Kullback–Leibler divergence, guiding the local model’s optimization to align with the private model’s objectives. This one-way distillation facilitates local client personalization during FL training. However, as illustrated in Figure 1b, the one-way knowledge transfer limits the private model’s access to global information from other clients, which diminishes its generalization capability.

Inspired by Deep Mutual Learning (DML) [16] and federated mutual learning (FML) [11], we introduce a bidirectional distillation to allow both local and private networks to learn from each other during the training process. Specifically, mutual learning employs Kullback–Leibler divergence to align the probability estimates of the two related networks. This bidirectional distillation can be adjusted by cross-referencing the experience information of local and private models, preventing overfitting due to the excessive smoothing of model training, and thus enhancing the generalization of the model. This strategy makes up for the shortcomings brought by one-way distillation so that the private model can learn the global knowledge contained in the local model while enhancing the personalization of the local client model. The mechanism of bidirectional distillation in FL learning is shown in Figure 1c. However, these approaches [11] transfer knowledge with the target class, not fully utilizing the information of the non-target class, which hinders the convergence speed and personalization of local models [17].

Therefore, to solve this problem, we propose a novel federated learning paradigm called Bidirectional Decoupled Distillation for Heterogeneous Federated Learning (BDD-HFL). Firstly, we facilitate the transfer of private knowledge to the local model by introducing a private model to each local client. Through bidirectional distillation during each training iteration, we ensure that knowledge transfer occurs without compromising model accuracy. Secondly, we optimize both the local and private models with decoupled relative-entropy loss. Specifically, we decouple the logistics output of all models to target class and non-target class information, giving balanced weight to them, and then the Kullback–Leibler loss is used to optimize them respectively. This allows us to balance the effect of knowledge distillation across target and non-target classes, enhancing the overall effectiveness of the process. After that, we integrate our method into classical federated learning methods to demonstrate its effectiveness. Experimental results demonstrate that BDD-HFL outperforms other personalized approaches. It addresses the challenges posed by data heterogeneity while maintaining local personalization.

The main contributions of this paper are as follows:

- We design a trustworthy federated learning framework with bidirectional distillation for heterogeneous scenarios, allowing two local networks to learn from each other through their decoupled relative-entropy loss. This approach prevents the increasing divergence in optimization direction between the local and private models, a common issue in one-way distillation methods.
- We introduce decoupled knowledge distillation to balance the effect of knowledge distillation across target and non-target classes. This approach enhances cross-referencing information interaction and improves the convergence speed of both server and client models.
- We integrate our proposed framework into four classical federated learning methods to verify its superiority on the CIFAR-10, CIFAR-100, and MNIST datasets.

## 2. Related Work

### 2.1. Heterogeneous Federated Learning

FL work has emerged as a prominent research area in recent years. McMahan et al. first proposed FedAVG [2], which demonstrated the effectiveness of FL while preserving data privacy. FedAVG achieves this by updating the parameters of multiple clients through weighted average parameters. However, Li et al. [18] pointed out that FL currently faces several challenges, including expensive communication costs between the server and the client, system heterogeneity at the client, data distribution among clients, and privacy issues.

Numerous studies have shown that Non-IID data distribution leads to degraded performance of the aggregated model, drifting of the updated client model, and slow convergence. Various federated learning (FL) approaches have been proposed to address these issues. Some works focus on reducing the variance in client updates to accelerate convergence. FedProx [19] enhances model stability by adding proximal regularization to the client model, where the proximal term reduces client drift by making the updated local model parameters close to the global model. However, this approach does not account for the fact that the optimal point of the local empirical target is different from the global optimal point, leading to lower performance. Scaffold [20] customizes the gradient update of the personalized model to correct the client drift. FedDyn [21] proposes a dynamic regularizer for each client to align the global and local models and also speed up model convergence. FedDC [22] uses the idea of grafting to fill parameter gaps with learned local drift variables as a way to maintain consistency in parameter levels. FedDisco [23] adds the discrepancy between local and global category distributions as a beneficial and complementary indicator to aggregate weights. The main limitation of these approaches is that they ignore differences in the client model and ignore local model personalization, leading to suboptimal performance. Some other approaches (Tuor [24]; Hybrid-FL [25]) use simple strategies such as data sharing, which implies that the client shares part of its data with the server, but data-sharing-based approaches may violate FL privacy requirements [26]. Also for privacy security, shared data can be extracted by distillation [27] or generated by generative adversarial networks (GANs [28]). The other approaches [29,30] optimize the FL training convergence by utilizing a probabilistic selection scheme. Refs. [31,32] presents a task-specific FL platform while reducing the overall communication cost.

In addition to the client drift problem, Non-IID data will also cause personalization problems. The server model, trained using data from all clients, often fails to generalize well to each client’s individual data, resulting in insufficient local personalization.

### 2.2. Personalized Federated Learning

Personalized federated learning for clients with Non-IID data has attracted a lot of attention. Existing methods can be divided into three categories: (1) global fine-tuning model, (2) local personalized aggregation model, and (3) local personalized private model.

The global fine-tuning model builds personalized models for each client by customizing a trained global model: Wang et al. [33] use each client’s private data to fine-tune the global model to generate a personalized model for the client. The model mixing approach APFL [34] combines a global model with a local model to customize a personalized model for the client. However, these methods do not fully consider the difficulties faced in the Non-IID case. The local personalized aggregation methods focus on learning local models with some aggregation strategy: FedAMP [35] generates an aggregated model for each client using an attention-inducing function and personalized aggregation. PartialFed [6] constructs a new local model for each batch by learning a binary and layer-level aggregation strategy for each client, selecting parameters from either the global model or the local model. FedALA [8] adaptively aggregates the downloaded global model and local model towards the local objective on each client to initialize the local model before training in each iteration. FedAF [9] proposed a novel aggregation-free FL algorithm to let clients collaboratively learn by leveraging peer knowledge. APPLE [7] involves downloading multiple client models, which increases the communication overhead and raises privacy concerns, as data from other clients may be compromised. The local personalized model methods learn local private models to achieve local personalization via information distillation: pFedMe [12] learns an additional personalized model for each client with Moreau envelopes. Specifically, it formulates a new bilevel optimization problem designed for personalized federated learning (pFedMe). By using the Moreau envelope as a regularized loss function, it decouples the cross-entropy optimization of personalized models from the learning of the global model. This approach updates the global model similarly to standard FL algorithms such as FedAvg while simultaneously optimizing the personalized models with low complexity. In Ditto [13], each client learns its own additional personalized model by incorporating a proximal term, which fetches information from the downloaded global model.

The current distillation model [36] uses a large pre-trained network as a teacher to provide additional knowledge to a smaller network of students. Applying this approach in FL [37], although the local knowledge is constantly transmitted to the client, the client’s model changes with the server-side model, causing the optimization direction of the private model with the local model to be larger and larger, and the mutual information interaction will seriously affect the performance of the global model. Unlike one-way knowledge transfer observed in a teacher–student network, Deep Mutual Learning (DML) [16] allows two networks to learn from each other through relative-entropy optimization during the training process. This bidirectional distillation mechanism is well suited for addressing the limitations associated with one-way distillation in the context of federated learning personalization problems, facilitating local personalization while maintaining model performance. However, the approach does not fully consider the transferability of knowledge and does not take full advantage of the model.

Hence, this article primarily integrates the concept of bidirectional distillation into federated learning to address the challenge of inadequate local personalization in Non-IID scenarios. Subsequently, we delve into the distillation process within bidirectional distillation in depth, aiming to enhance the effectiveness of model knowledge transfer.

## 3. Methods

To tackle the challenges stemming from the catastrophic forgetting of local data, insufficient local personalization, and diminished model effectiveness due to data heterogeneity, we introduce a more adaptable federated learning approach in this section: Bidirectional Decoupled Distillation For Heterogeneous Federated Learning. This approach ensures local personalization while significantly enhancing the performance of global models.

### 3.1. Problem Definition

In conventional FL, there is a server and N clients. The server randomly initializes the global model and sends the global model to selected clients, and each client copies the model and trains it using its private data. Subsequently, the trained model parameters are uploaded to the server, where they are aggregated and subjected to a weighted average. The server then transmits the aggregated parameters back to the clients, repeating this iterative process until model training converges. The overarching training objectives are delineated below:

(1) $$w_{t}=arg\min_{w}F\left(w\right)=\sum_{k=1}^{K}\frac{n_{k}}{n}f_{k}\left(w\right)$$

The aim is to obtain a server-side model parameter $w_{t}$. $f_{k}\left(w\right)$ denotes the local cross-entropy loss function of the client, *K* represents the total number of clients, *n* represents the number of samples of all clients, and $n_{k}$ represents the amount of private data of client *k*. The process of server-side distribution is $w\to w^{k}$, where clients copy the server-side parameters as local model parameters. The whole process of server-side model aggregation is as follows:

(2) $$w_{t+1}=\sum_{k=1}^{K}\frac{n_{k}}{n}w_{t}^{k}$$

Its $w_{t}^{k}$ represents the model parameters of local client *k* after the *t*th round of training. This signifies that the server sequentially aggregates the model parameters from the client side, weighted by the data, to produce the model parameters for the server side in the subsequent round.

Rather than solely addressing the conventional federated learning (FL) problem, we obtain local client-personalized models while the global model converges to the optimal level.

### 3.2. Framework

**Figure 2:**
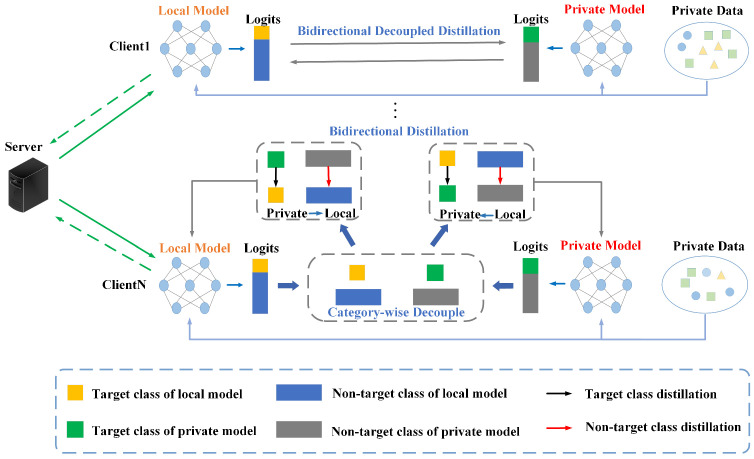
Each client in the BDD-HFL method contains two models: the local model and the private model, where the local model replicates the global model, and the private model is responsible for multiple rounds of decoupled Deep Mutual Learning with the local model. The grey line denotes bidirectional decoupled distillation, blue line denotes local update, and green line denotes server update. In scenarios where clients have heterogeneous data distributions, the logits of both local and private models are decoupled into target and non-target classes during local training. Bidirectional decoupled distillation facilitates mutual learning between the local and private models by allowing them to distill information from each other, focusing separately on target and non-target classes. This process refines both models. After each major round of local training, the refined local models are aggregated and uploaded to the server.

The whole framework of Bidirectional Decoupled Distillation For Heterogeneous Federated Learning (BDD-HFL) is shown in Figure 2. The server first delivers the global model to every client as the local model, including $Client_{1}$ to $Client_{n}$. The additional private models act as the personalized model. For local training, every client performs training updates of the two local and private models. Taking $Client_{n}$ in Figure 2 as an example, the logits of the local model are decoupled into the target class (the yellow square) and non-target classes (the blue rectangle) while the logits of the private model are decoupled into the target class (the green square) and non-target classes (the grey rectangle). In bidirectional distillation between private and local models, each model distills information related to both target and non-target classes from the other. After several rounds of local updates, the client uploads the refined local model to the server, contributing to the generation of a new global model.

### 3.3. Bidirectional Decoupled Knowledge Distillation

One-way distillation from private to local is developed by relative-entropy loss, named Kullback–Leibler (KL) divergence loss. The relative-entropy loss is described as the formula below:

(3) $$\mathrm{KL}\left(p_{pri}∥p_{loc}\right)=\sum_{i=1}^{M}p_{pri}^{i}\log\frac{p_{pri}^{i}}{p_{loc}^{i}}$$

where $p_{loc}$ and $p_{pri}$ are the logits of the local and private models, while *M* is the class number. $KL(p_{pri}∥p_{loc})$ is used to distill knowledge from the private model to the local model. However, the traditional one-way distillation process limits the effective transfer of global information and hinders comprehensive knowledge exchange. Additionally, Kullback–Leibler (KL) loss tends to prioritize the target class, often overlooking the significance of non-target classes, which restricts the flexibility and learning capacity of both models. To address these issues, we introduce a novel bidirectional distillation method with decoupled relative-entropy loss for both local and private models. Unlike the conventional approach, our distillation process operates in two directions: from the local model to the private model, and from the private model to the local model. We structure the knowledge exchange through decoupled distillation into two components, target class (TC) and non-target class (NC) knowledge distillation, weighted appropriately to balance their contributions. Here, we define *t* as the index of the target class. The Formula (3) can be rewritten as:

(4) $$\mathrm{KL}\left(p_{pri}∥p_{loc}\right)=p_{pri}^{t}\log\frac{p_{pri}^{t}}{p_{loc}^{t}}+\sum_{i=1,i\neq t}^{M}p_{pri}^{i}\log\frac{p_{pri}^{i}}{p_{loc}^{i}}$$

Hence, the decoupled relative-entropy loss (DREL) can be described as follows:

(5) $$\mathrm{DREL}\left(p_{pri}∥p_{loc}\right)=\begin{array}{c} \alpha \underset{︸}{p_{pri}^{t}\log\left(\frac{p_{pri}^{t}}{p_{loc}^{t}}\right)}\\ TC \end{array}+\begin{array}{c} \underset{︸}{\beta \sum_{\begin{array}{c} i=1,i\neq t \end{array}}^{M}p_{pri}^{i}\log\left(\frac{p_{pri}^{i}}{p_{loc}^{i}}\right)}\\ NC \end{array}$$

where the α and β balance the importance of the target class and non-target class distillation for more efficient knowledge transfer.

Then, we add the decoupled knowledge distillation to the federated process and rewrite the joint loss function for the private model as well as the local model as follows:

(6) $$\begin{array}{c} L_{loc}=L_{C_{loc}}+\mathrm{DREL}\left(p_{pri}∥p_{loc}\right) \end{array}$$

(7) $$\begin{array}{c} L_{pri}=L_{C_{pri}}+\mathrm{DREL}\left(p_{loc}∥p_{pri}\right) \end{array}$$

where $L_{C_{pri}}$ and $L_{C_{loc}}$ denote the cross-entropy loss, $DREL(p_{pri}∥p_{loc})$ is the decoupled relative-entropy loss from the private model to the local model, and $DREL(p_{loc}∥p_{pri})$ is the relative-entropy loss from the local to the private model. The direction of our knowledge transfer between the local model and the private model is bidirectional, where the local model transfers the server-side knowledge to the private model and obtains feedback from it, while both models are trained using private data with joint optimization on $DREL(p_{pri}∥p_{loc})$ and $DREL(p_{loc}∥p_{pri})$. The effectiveness of the knowledge transfer can be improved while addressing local personalization, which improves the model’s performance.

### 3.4. Training Pipeline

In Algorithm 1, we show the algorithm pseudo-code of the process of BDD-HFL. We denote the global model as global, total number of clients as *K*, and communication rounds as *T*. Firstly, the server initializes the global model. In each round *t*, *k* clients are randomly selected, obtaining the global model global as their local model ${local}_{t}^{k}$, then executing the ClientUpdate process and obtaining the updated local model ${local}_{t+1}^{k}$. On the client side, we first decouple the logits of the local model to the target class and non-target class. After that, we implement the bidirectional distillation, optimized with decoupled relative-entropy loss: ${local}_{t}^{k}\leftarrow {private}_{t}^{k}$ and ${private}_{t}^{k}\leftarrow {local}_{t}^{k}$. Finally, the global is updated by the weighted aggregate ${local}_{t+1}^{k}$. Each client pushes its trained local model to the server, which weights and averages these local models to obtain a new global model. The whole process is repeated until the global model converges.

As illustrated in Algorithm 1, our proposed BDD-HFL shares the logits (soft labels) of private samples rather than model parameters. This design allows the federated distillation framework to support heterogeneous local models and reduces the risk of white-box privacy attacks as supported by the findings in [38,39]. The bidirectional distillation process can also be interpreted as introducing noise to the global parameters during uploading and downloading, thereby enhancing privacy protection. Moreover, the distillation occurs dynamically between the local client and the private model without involving the server. This separation breaks the connection between the server and the private model, further increasing the difficulty of attacks that rely on shared model parameters.    
**Algorithm 1:** BDD-HFL**1** **Server:****2** Initialize the global model: global, total number of clients: *K*, communication rounds *T***3** **for** *each round t = 1, 2, …, T* **do**
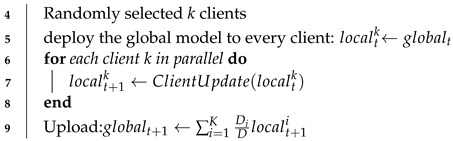
**10** **end**(**11** **ClientUpdate:****12** Initialize the local model ${local}_{t}^{k}$ and the private model ${pri}_{t}^{k}$, the local epoch: *E*,**13** distributing: ${local}_{t}^{k}\leftarrow global$**14** **for** *each epoch e = 1, 2, …, E* **do**
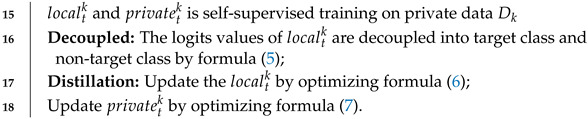
**19** **end****20** Get ${local}_{t+1}^{k}$ and upload it to sever

## 4. Results

In this section, we focus on verifying the effectiveness of the BDD-HFL method across various existing federated learning methods, as well as testing the performance on different natural datasets. In addition, we compare the experimental results of one-way distillation KD, bidirectional distillation FML, and our method on some baselines, for instance, FedAvg [2], FedProx [19], FedDyn [21], FedDC [22], and FedDisco [23].

In the above, we mainly evaluate the trustworthiness of our proposed method by evaluating its robustness [40] and fairness [41] in different heterogeneous scenarios. To evaluate robustness, we examine the method’s generalization capabilities in Heterogeneous Federated Learning (HFL) under various scenarios, including IID, Non-IID, and unbalanced data distributions, using average classification accuracy as a metric. This evaluation is crucial because each client possesses different local data heterogeneity, and the aggregated global model may struggle to capture individual data patterns effectively. In contrast, we assess fairness in HFL by evaluating client selection with accuracy metrics. Here, fairness is measured by considering scenarios where only a fraction of clients are selected to participate in the training process of each round. This evaluation ensures that the method performs equitably across different clients, regardless of their selection frequency or data distribution.

### 4.1. Dataset

We conduct comparisons across three datasets, including the natural datasets cafir10, cafir100 [42], and MNIST [43]. We follow the same experimental setting with FedDC [22]. We classify the data into two primary distributions: IID and Non-IID. In the IID setting, the training data are evenly and randomly distributed across all clients, with each category equally distributed among clients. Conversely, in the Non-IID setting, we divide the data into two distributions according to the Dirichlet distribution [44]: D1 = 0.6 and D2 = 0.3. D2 exhibits greater heterogeneity compared to D1. Respectively, we adopt LeNet convolutional neural networks [43] for CIFAR-10 and CIFAR-100 datasets, and a fully connected network (FCN) [45] for the MNIST dataset.

### 4.2. Baseline and Hyper-Parameter Settings

Furthermore, we evaluate the efficacy of our method by integrating it with various federated learning methods, including FedAvg [2], FedProx [19], FedDyn [21], FedDC [22], and FedDisco [23]. These methods primarily address the issue of client-side model drift caused by data heterogeneity, but they do not explicitly consider the problem of insufficient personalization of local models due to the aggregation-induced catastrophic forgetting of knowledge. Hence, we validate the effectiveness of our approach across these five baselines.

Most parameters continue to extend the settings of the FedDC, with the difference that we add decoupled bidirectional knowledge distillation between the private and local models during the learning process, and the local model interacts with the server model, while the private model is kept locally from the beginning to the end. We initialize the private model, local model, and server-side model to be identical. For the distillation hyperparameters, we set λ=0.5 and μ=0.5 for both the local model and private model. Additionally, to emphasize non-target class knowledge, we set the distillation weight of the target class knowledge to α=1.0 and the distillation weight of the non-target class knowledge to β=8.0. We utilize the SGD algorithm as the local optimizer for all methods. To ensure consistency, we set the batch size to 50 during the local training phase, with each round consisting of five local epochs. The initial learning rate is set to 0.1, with a decay rate of 0.998. Respectively, we run federated learning for 600 rounds on MNIST and 1000 rounds on CIFAR-10 and CIFAR-100 datasets. We set the number of clients to 500 and 100 and divide them into full participation and 15% partial participation. We set the penalty coefficient of FedDC and FedDisco as 0.01 on the CIFAR10 and CIFAR100 datasets and 0.1 on the MNIST datasets.

### 4.3. Results and Analysis

We integrate our BDD-HFL method into both classic federated learning (FL) models and the latest FL methods, conducting numerous comparative experiments to assess improvements in accuracy. Additionally, we design various degrees of data heterogeneity to evaluate the model’s robustness and applicability. By comparing the performance before and after incorporating the BDD-HFL method, we validate its effectiveness in enhancing model accuracy and personalization capabilities, with the primary validation being based on the final model’s performance on the server side. Furthermore, we compare our BDD-HFL method with bidirectional distillation FML and one-way distillation KD.

**Better performance of BDD-HFL.** We keep the local model personalized by adding a private model to the client and passing local knowledge to the local model during the iteration process. In Table 1 and Table 2, we present a comparison of the experimental results obtained by incorporating one-way distillation KD, bidirectional distillation DML, and our BDD-HFL method into the latest federated learning approaches, FedAvg, FedDyn, FedProx, FedDC, and FedDisco. It is found that among the three datasets, the addition of both the KD and DML methods impair the performance of FedDC, and the KD effect is worse, which is 84.80% and 85.39% for partial participation and full participation in setting one. In contrast, the addition of our BDD-HFL method is 86.83% and 86.65% for partial participation and full participation in setting one. As a result, it significantly enhances the performance of FedDC, particularly in scenarios with multiple heterogeneous effects across different datasets. For the four kinds of data distribution in Setting 1, our method, combined with FedDisco, achieves an accuracy of 86.35% on the setting of CIFAR10-IID, 85.82% on the setting of CIFAR10-D1, 84.88% on the setting of CIFAR10-D2, and 86.20% on the setting of CIFAR10-unbalance in 100 clients’ partial participation. These results maintain a similar accuracy, which proves that our method is robust to different data heterogeneities and data unbalance situations. We analyze that KD and FML do not necessarily enhance heterogeneous FL, and heterogeneous data characteristics need to be considered. Our method considers this issue by decoupling the logits values into target class and non-target class distillation, which are guided by decoupled relative-entropy loss. The relative-entropy loss is measured by assigning greater weight to non-target classes compared to target classes. By focusing on the importance of non-target class knowledge, local and private models in the client can learn multi-class information, enhancing the effectiveness of knowledge transfer. This approach effectively maintains local personalization while preserving and even improving the performance of the global model.

**Generalization to client structure.** In Table 1 and Table 2, we validate the results of the experiments in two different settings: partial participation and full participation of the clients. For partial participation setting (100 clients), we test three datasets, each with four different ways of distributing the data. On top of this, we conduct a comparative analysis of the efficacy between incorporating bidirectional distillation, one-way distillation, and our BDD-HFL approach, against traditional federated learning methods. The results show that BDD-HFL on cafir10 always shows the best performance and is better suited to traditional federated learning methods, while the bidirectional distillation learning approach shows worse results. Similarly, on cafir100, our method again proves to have better stability and applicability. For MNIST data, our method is still effective in FedDyn and FedAvg, and has no serious impact on the other methods, and it is worthwhile to say that, for the two heterogeneous conditions of MNIST-D1 and MNIST-D2, we can also show the effectiveness of our method in solving the problem of insufficient personalization caused by the heterogeneous data.

For full participation setting (500 clients), the results show that our BDD-HFL method is also effective in large-scale distributed settings, and our method also improves 0.98% on CIFAR10-IID and 3.93% on CIFAR100-IID in the latest federated learning method FedDC. We validate the experimental results in the case of full client participation and find that our method improves the performance of the model regardless of the total number of clients as well as the number of client participants. The experiments show that our method effectively compensates for the lack of local personalization in classical federated learning and improves the performance of the model. The unique feature of BDD-HFL is the introduction of a novel bidirectional distillation decoupling idea, which adds a private model locally so that the local model can learn more extra knowledge while approaching the optimization direction of the private model no matter how many clients there are, thus making up for the poor local performance caused by heterogeneous data on the client.

**Robustness to client sampling.** From Table 1, we find that, for the case of the full participation of 100 clients, the accuracy of our method added on the FedDC on CIFAR10-IID is 86.65%, but for the case of the partial participation of 100 clients, the accuracy rises to 86.83%. Combined with the results of the other experiments in Table 2, we can see that the decision on the amount of client participation does not have a great impact on the experimental results, and the partial participation of the clients can still maintain a similar level of accuracy to the full participation of the clients. Therefore, based on the above performance, our method does not affect the elasticity of the client sampling, which shows our method performs well on different types and numbers of clients. This result proves the robustness and fairness of our method. It is only through the private model that the network will refer to the experience of the private model network to adjust its learning during the training process, and ultimately be able to converge to a smoother point of the smallest value so that it has better generalization performance, thus improving the performance of the global model, and it will not be interrupted due to the interruption of the training of the model.

**Figure 3:**
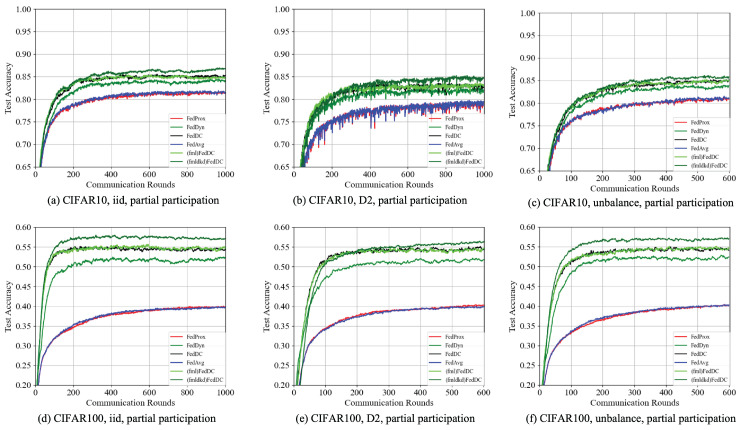
Convergence plots of BDD-HFL plus on FedDC and DML plus on FedDC and other baselines in different settings for the CIFAR10 and CIFAR100 datasets with IID, D2 Non-IID (Dirichlet-0.3), and imbalanced data with 100 clients participating. (**a**–**c**) represent partial participation in CIFAR10 training. (**d**–**f**) represent full participation in CIFAR 100 training.

**Convergence on heterogeneous data.** Due to the characteristics of wide data distribution, the model convergence speed is often slow. In Figure 3, we compare the convergence graphs (a and b, and d and e) to illustrate the different convergence speeds and model accuracies for various data distributions. The results indicate that convergence under IID data distribution is faster than under Non-IID conditions, and model accuracy is also higher with IID data.

When applying our method, BDD-HFL, on top of FedDC, we observe significant improvements in different scenarios of data heterogeneity and client numbers. Our method consistently outperforms other methods not utilizing BDD-HFL in terms of both convergence speed and model accuracy. This includes superior performance compared to FML added to FedDC. Even in heterogeneous setups, the convergence graphs clearly visualize the enhancements our method brings, demonstrating its effectiveness in improving both convergence speed and model accuracy.

## 5. Conclusions

In this work, we address the issue of insufficient local personalization caused by data heterogeneity by proposing Bidirectional Decoupled Distillation For Heterogeneous Federated Learning (BDD-HFL). BDD-HFL leverages the interaction between the local model and a private model through decoupled relative-entropy knowledge distillation, enabling a more flexible learning process. Extensive experiments on various categorized datasets demonstrate that our BDD-HFL approach outperforms other federated learning methods.

**Limitations.** Our method integrates an additional private model into each client, and knowledge distillation approaches in FL typically assume that both the private and local models have access to the same local training data. This assumption increases the risk of information leakage and adds to the computational cost during the training phase. In contrast, Data-Free Knowledge Distillation (DFKD) transfers knowledge by generating synthetic data, thereby eliminating the need to access the original data. By further analyzing DFKD strategies, the above issues may be tackled, which is left as our future work. Furthermore, heterogeneous scenarios involve heterogeneous multi-task clients or heterogeneous client architectures. In the future, we will explore various federated learning scenarios to evaluate our proposed method.

## Figures and Tables

**Table 1:** Top 1 test accuracy (%) for IID, Non-IID, and unbalanced data with 100 and 500 clients partially engaged (15%). The comparison method is to add KD, DML, and BDD-HFL implemented with FedAvg [2], FedProx [19], FedDyn [21], FedDC [22], and FedDisco [23] five baselines. Bold numbers indicate the best results.

Methods	FedAvg	+KD	+FML	+BDD-HFL	FedProx	+KD	+FML	+BDD-HFL	FedDC	+KD	+FML	+BDD-HFL	FedDyn	+KD	+FML	+BDD-HFL	FedDisco	+KD	+FML	+BDD-HFL
**Setting 1**	100 clients partial participation																			
CIFAR10-IID	81.67	80.79	81.26	**82.41**	**82.16**	81.78	81.57	82.02	85.71	84.80	84.94	**86.83**	84.50	83.61	83.96	**85.20**	84.81	85.06	85.28	**86.35**
CIFAR10-D1	81.05	80.25	80.77	**82.34**	81.32	80.65	80.81	**82.31**	84.77	84.26	84.33	**85.90**	84.10	82.55	83.17	**84.60**	84.40	84.36	84.38	**85.82**
CIFAR10-D2	79.77	79.03	80.19	**81.67**	79.84	79.57	79.62	**82.23**	84.58	82.97	83.56	**85.03**	82.30	81.33	82.37	**83.33**	83.49	82.97	83.17	**84.88**
CIFAR10-unbalance	81.68	81.43	81.53	**81.87**	**81.88**	81.41	81.24	81.46	85.35	84.64	84.94	**85.81**	84.30	84.18	84.30	**85.12**	84.90	84.76	84.84	**86.20**
CIFAR100-IID	40.80	42.09	42.11	**44.47**	40.67	40.51	41.91	**42.92**	55.40	54.53	54.56	**56.94**	51.20	50.02	50.74	**52.13**	54.04	54.04	54.51	**57.37**
CIFAR100-D1	41.76	41.78	42.48	**45.78**	41.83	40.68	42.23	**47.50**	54.65	54.01	54.46	**57.00**	**51.75**	48.83	50.19	51.50	53.84	53.62	54.25	**57.04**
CIFAR100-D2	41.81	42.13	42.98	**47.24**	41.84	41.12	42.57	**49.98**	53.91	52.89	53.11	**56.39**	**51.13**	47.71	49.44	50.64	53.93	53.44	53.67	**56.39**
CIFAR100-unbalance	40.90	43.19	42.16	**43.27**	41.05	41.33	40.55	**42.94**	55.27	53.72	53.79	**56.45**	51.01	50.16	**51.03**	50.39	54.21	54.15	54.67	**56.80**
MNIST-IID	98.15	98.12	97.99	**98.20**	98.11	98.19	**98.19**	98.08	98.47	98.42	98.45	**98.49**	98.38	98.35	98.30	**98.39**	98.39	**98.53**	98.42	98.48
MNIST-D1	98.13	98.05	97.99	**98.20**	98.12	98.19	98.14	**98.22**	**98.49**	98.45	98.42	98.43	98.30	98.26	98.21	**98.35**	98.36	**98.49**	98.40	98.46
MNIST-D2	98.00	97.91	98.03	**98.21**	98.04	98.02	98.01	**98.05**	98.40	98.40	98.35	**98.46**	98.30	98.25	98.12	**98.44**	98.28	98.41	98.46	**98.47**
MNIST-unbalance	98.15	98.16	98.02	**98.16**	98.13	98.10	**98.18**	98.10	**98.53**	98.49	98.41	98.16	**98.34**	98.30	98.31	98.32	98.37	98.42	98.40	**98.56**
**Setting 2**	500 clients partial participation																			
CIFAR10-IID	73.26	73.09	73.26	**75.74**	72.58	72.66	72.90	**75.03**	84.19	82.26	81.70	**85.17**	82.49	81.12	81.16	**82.77**	74.34	79.84	79.36	**81.78**
CIFAR100-IID	27.36	**28.46**	27.93	27.36	26.50	27.23	**27.47**	24.52	50.61	54.53	43.38	**54.54**	44.11	44.48	44.25	**40.58**	41.17	36.90	43.80	**53.02**

**Table 2:** Top 1 test accuracy (%) for IID, Non-IID, and unbalanced data with 100 and 500 clients fully engaged. The comparison method is to add KD, DML, and BDD-HFL implemented with FedAvg [2], FedProx [19], FedDyn [21], FedDC [22], and FedDisco [23] five baselines. Bold numbers indicate the best results.

Methods	FedAvg	+KD	+FML	+BDD-HFL	FedProx	+KD	+FML	+BDD-HFL	FedDC	+KD	+FML	+BDD-HFL	FedDyn	+KD	+FML	+BDD-HFL	FedDisco	+KD	+FML	+BDD-HFL
**Setting 1**	100 clients all participation																			
CIFAR10-IID	82.16	81.84	81.53	**82.76**	81.85	81.76	81.80	**82.58**	86.18	85.39	85.38	**86.65**	85.26	82.48	84.59	**85.84**	85.49	85.06	85.71	**87.07**
CIFAR10-D1	80.42	80.89	80.86	**82.36**	80.70	80.43	80.75	**82.83**	85.64	85.09	85.30	**86.36**	85.26	84.17	84.50	**85.69**	85.11	85.16	85.49	**85.75**
CIFAR10-D2	79.14	80.82	80.92	**81.82**	40.93	40.27	41.56	**48.44**	84.32	83.59	83.69	**84.83**	84.14	83.37	82.97	**84.17**	83.98	84.30	84.13	**84.95**
CIFAR10-unbalance	**86.31**	81.80	81.67	82.49	81.90	81.58	81.71	**82.56**	86.31	85.41	85.74	**86.85**	85.68	84.50	84.93	**86.15**	85.55	85.84	85.81	**87.02**
CIFAR100-IID	39.68	41.81	41.70	**43.15**	40.39	38.79	39.58	**42.94**	55.52	53.99	54.61	**57.11**	52.07	51.68	51.46	**53.53**	53.57	54.67	54.36	**56.95**
CIFAR100-D1	40.48	42.09	42.38	**44.07**	40.15	40.24	40.84	**45.55**	55.34	53.76	54.66	**57.94**	**52.84**	51.62	51.84	52.76	54.68	53.43	55.13	**57.32**
CIFAR100-D2	40.11	43.14	43.27	**43.56**	40.93	40.27	41.56	**48.44**	54.86	53.76	54.63	**56.89**	51.89	50.40	50.67	**53.01**	53.90	53.83	54.15	**57.16**
CIFAR100-unbalance	40.03	43.31	42.25	**44.12**	39.93	40.08	40.76	**41.82**	55.69	53.87	54.55	**57.06**	**52.81**	51.70	52.17	52.55	54.18	54.77	54.24	**57.15**
MNIST-IID	98.12	98.49	**98.50**	98.45	98.12	**98.21**	98.19	98.14	98.45	98.42	98.44	**98.45**	98.51	**98.72**	98.66	98.63	98.33	98.46	98.42	**98.48**
MNIST-D1	98.09	98.43	**98.48**	98.31	98.05	98.15	98.11	**98.15**	98.48	98.45	98.45	**98.53**	98.44	**98.72**	98.67	98.55	98.39	98.44	98.46	**98.49**
MNIST-D2	97.98	98.38	98.35	**98.39**	97.96	98.06	**98.12**	98.10	98.51	98.44	98.45	**98.58**	98.46	**98.70**	98.57	98.64	98.40	98.49	**98.52**	98.46
MNIST-unbalance	98.12	**98.49**	98.37	98.35	98.10	98.26	**98.31**	98.18	98.46	98.48	98.43	**98.48**	98.60	98.62	**98.73**	98.69	98.43	98.46	98.46	**98.48**
**Setting 2**	500 clients all participation																			
CIFAR10-IID	73.43	72.86	73.01	**75.63**	72.77	71.57	72.53	**75.17**	84.93	85.39	83.93	**86.36**	84.07	83.38	83.43	**84.73**	82.20	82.06	84.25	**85.40**
CIFAR100-IID	26.03	**27.95**	27.75	27.86	**28.22**	26.48	27.53	27.04	54.25	38.86	47.89	**56.26**	50.22	**51.95**	51.75	51.80	50.05	38.60	49.46	**56.38**

## Data Availability

We use public CIFAR datasets to evaluate the performance of proposed method. The CIFAR-10 and CIFAR-100 public datasets can be freely downloaded from the web page at https://www.cs.toronto.edu/~kriz/cifar.html, accessed on 7 October 2022. The MNIST dataset can be freely downloaded from the website https://opendatalab.com/MNIST, accessed on 12 June 2022. The source code of our BDD-HFL approach is available at https://github.com/drafly/BDKFD.git.
